# Eukaryotic Translation Initiation Factor 4 Gamma 1 (eIF4G1) is upregulated during Prostate cancer progression and modulates cell growth and metastasis

**DOI:** 10.1038/s41598-018-25798-7

**Published:** 2018-05-10

**Authors:** Praveen Kumar Jaiswal, Sweaty Koul, Prakash S. T. Shanmugam, Hari K. Koul

**Affiliations:** 1 Department of Biochemistry and Molecular Biology, LSU Health Sciences Center, Shreveport, 1501 Kings Highway, LA 71130 USA; 2 Department of Urology, LSU Health Sciences Center, Shreveport, 1501 Kings Highway, LA 71130 USA; 3 Overton Brooks Veterans Administration Medical Center, Shreveport, LA USA; 4 Feist Weiller Cancer Center, Shreveport, 1501 Kings Highway, LA 71130 USA

## Abstract

eIF4G1, a critical component of the eIF4F complex, is required for cap-dependent mRNA translation, a process necessary for tumor growth and survival. However, the role of eIF4G1 has not been evaluated in Prostate Cancer (PCa). We observed an increased eIF4G1 protein levels in PCa tissues as compared to normal tissues. Analysis of the TCGA data revealed that eIF4G1 gene expression positively correlated with higher tumor grade and stage. Furthermore, eIF4G1 was over-expressed and or amplified, in 16% patients with metastatic PCa (SU2C/PCF Dream Team dataset) and in 59% of castration-resistant prostate cancer (CRPC) patients (Trento/Cornell/Broad dataset). We showed for the first time that eIF4G1 expression was increased in PCa and that increased eIF4G1 expression associated with tumor progression and metastasis. We also observed high protein levels of eIF4G1 in PCa cell lines and prostate tissues from the TRAMP model of PCa as compared to normal prostate cell line and prostate tissues from the wild type mice. Knockdown of eIF4G1 in PCa cells resulted in decreased Cyclin D1 and p-Rb protein level, cell cycle delay, reduced cell viability and proliferation, impaired clonogenic activity, reduced cell migration and decreased mRNA loading to polysomes. Treatment with eIF4G complex inhibitor also impaired prostasphere formation. eIF4G1 knockdown or treatment with eIF4G complex inhibitor sensitized CRPC cells to Enzalutamide and Bicalutamide. Our results showed that eIF4G1 plays an important role in PCa growth and therapeutic resistance. These data suggested that eIF4G1 functions as an oncoprotein and may serve as a novel target for intervention in PCa and CRPC.

## Introduction

Prostate cancer is the second most frequently diagnosed malignancy in men in the USA^1^. Conventional therapies provide a high percentage of the cure for patients with localized prostate cancer, but there is no cure once the disease has spread beyond the prostate and once it fails to respond to androgen deprivation therapies^2^. Metastatic castration-resistant prostate cancer (CRPC) is estimated to result in about 26,730 deaths in 2017 in the USA^1^. There is an urgent and unmet need for identification and characterization of new molecular targets for efficient diagnosis and development of novel therapeutic options in PCa.

Cap-dependent translation is essential to maintain high protein synthesis and translation of specific mRNAs that are responsible for various tumorigenic properties in cancer cells. Translational control occurs predominately during a rate-limiting, initiation step which is subjected to extensive regulation^3,4^ and is governed by cap-binding complex, eukaryotic initiation factor 4 F (eIF4F) which comprises cap-binding protein eIF4E, eIF4A (helicase) and eIF4G (scaffolding protein). The eIF4F complex recruits ribosomes to mRNA such that the 5′ untranslated region (5′ UTR) can be scanned by ribosomes in search of an initiation codon^4^.

An interaction between eIF4G and eIF4E is crucial for the formation of the eIF4F complex and initiation of cap-dependent translation^5^. The eIF4G family comprises three isoform eIF4G1, eIF4G2 and eIF4G3^6^ among which eIF4GI is the major isoform (>85%)^7^. eIF4G1 and eIF4G3 isoform are involved in the cap-dependent translation, while eIF4G2 is associated with IRES-dependent translation in cells^6,8^.

The eIF4F complex has been shown to play an important role in oncogenesis^9,10^. It’s known that interaction of eIF4G1-eIF4E not only governs the protein synthesis but also its quality and thus contribute to the cell phenotype and function^11^. Recent reports suggest that eIF4G1 plays an important role in the tumorigenesis and is over-expressed in several solid tumors^12–19^. Moreover, the chromosomal location of eIF4G1 (3q27.1) is amplified in PCa patients^20^. However, the role of eIF4G1 has not been evaluated in PCa.

In the present study, we evaluated the expression of eIF4G1 in prostate cancer samples, analyzed eIF4G1 expression in multiple prostate cancer cohorts and investigated the functional role of eIF4G1 using cell culture model systems. Our results, presented herein, demonstrate for the first time that increased eIF4G1 expression in PCa was associated with tumor progression. Our results further showed that eIF4G1 enhanced cell proliferation and cell migration and is required for clonogenic activity. eIF4G1 knockdown sensitized CRPC cells (C4-2B cells) to Enzalutamide and Bicalutamide. Moreover, treatment with eIF4G inhibitor impaired prostasphere formation and further impairs clonogenic activity in combination with Enzalutamide in C4-2B cells. These data suggest that eIF4G1 may function as an oncoprotein and may serve as a novel target for intervention in PCa and CRPC.

## Results

### eIF4G1 is over-expressed in multiple clinical cohorts

**Figure 1:**
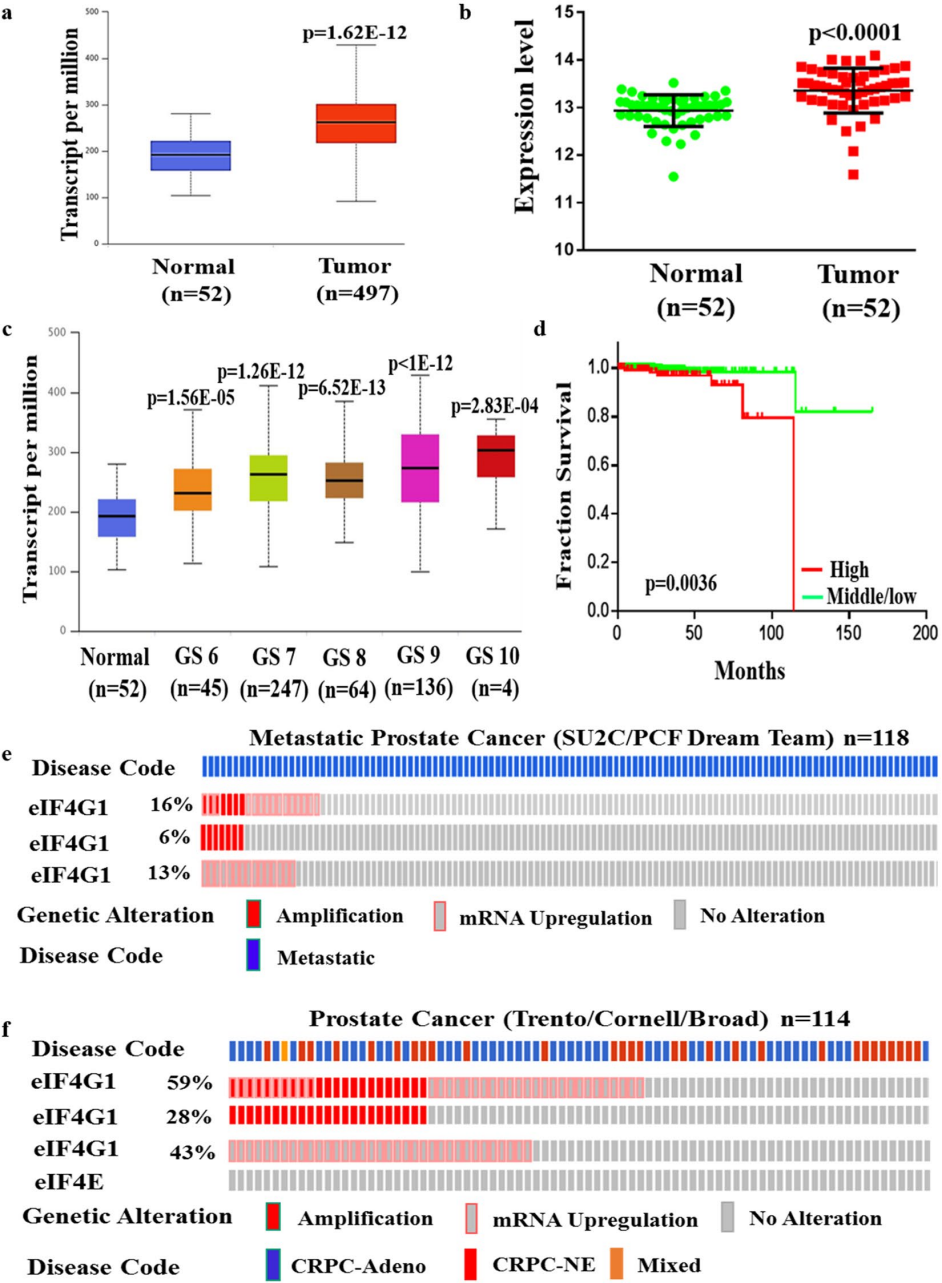
eIF4G1 is over-expressed in multiple clinical cohorts of PCa: Analysis of eIF4G1 expression in PCa samples from different databases: (**a**) mRNA levels of eIF4G1 in Normal vs prostate tumor samples from TCGA dataset. (**b**) mRNA levels of eIF4G1 in Paired Normal vs prostate tumor samples from TCGA dataset. (**c**) A Graded increase in eIF4G1 with increasing Gleason Score (GS) (TCGA). (**d**) High expression of eIF4G1 decreases survival probability (TCGA data set, time in months). (**e**) eIF4G1 is amplified and overexpressed in 16% patients with Metastatic Prostate Cancer dataset. (**f**) eIF4G1 is amplified and overexpressed in 59% patients in CRPC-Neuroendocrine Prostate Cancer dataset, while no change is observed in eIF4E. p-values are indicated as *<0.05, **<0.01, ***<0.001.

First, we analyzed data from TCGA, which includes 497 primary PCa samples and 52 normal prostate tissues. Our result showed that mRNA level of eIF4G1 in primary tumor was significantly higher compared to normal prostate tissue (p = 1.62E-12) (Fig. 1a). Results of our paired sample (n = 52) analysis of eIF4G1 expression from TCGA database (Fig. 1b) also revealed higher expression of eIF4G1 in PCa tissues compared to adjacent normal tissues. Moreover we observed a graded increase in eIF4G1 mRNA expression with increasing tumor grade (Gleason Score) with a significant p-value with the comparison between normal to Gleason Score (GS) 6 (p = 1.56E-05), GS 7 (p = 1.62E-12), GS 8 (p = 6.52E-13), GS 9 (p < 1E-12) and GS 10 (p = 2.83E-04) (Fig. 1c). Our analysis of survival data revealed that PCa patients with high eIF4G1 expression had lower median survival (Approx. 9.58 years) compared to the patient with low/Medium eIF4G1 expression (Approx. 13.69 years) (p < 0.0036) (Fig. 1d). Another important driver of cap-dependent translation, eIF4E has been associated with the aggressiveness of cancer. However, our analysis of eIF4E mRNA expression in TCGA database revealed an insignificant difference for eIF4E expression between normal prostate and tumor samples as well as with tumor grade stage except with Gleason score 6 patients where eIF4E expression was significantly (p = 2.79E-03) decreased (Fig. S1a,b).

Furthermore, we also analyzed the mRNA expression data for eIF4G1 in available clinical data set for patients with metastatic PCa (SU2C/PCF Dream Team; n = 118) and CRPC/neuroendocrine (Trento/Cornell/Broad; n = 114) phenotype through the cBioPortal web server using Z-score threshold of ±2. We observed that 16% and 59% of patients have genetic alteration such as amplification and mRNA up-regulation in eIF4G1 in metastatic PCa and CRPC/Neuroendocrine PCa, respectively. Metastatic PCa dataset showed that 6% patients have amplification and 13% patients have mRNA up-regulation in eIF4G1 gene (Fig. 1e) while CRPC/Neuroendocrine PCa dataset revealed that 28% patients showed amplification in eIF4G1 gene and 43% patients show mRNA up-regulation of eIF4G1 (Fig. 1f). Interestingly we found that there was no change in eIF4E in patients with CRPC/Neuroendocrine PCa phenotype. Overall, these data demonstrate that the transcriptional up-regulation in the eIF4G1 of the cap-dependent translational initiation complex was associated with PCa.

**Figure 2:**
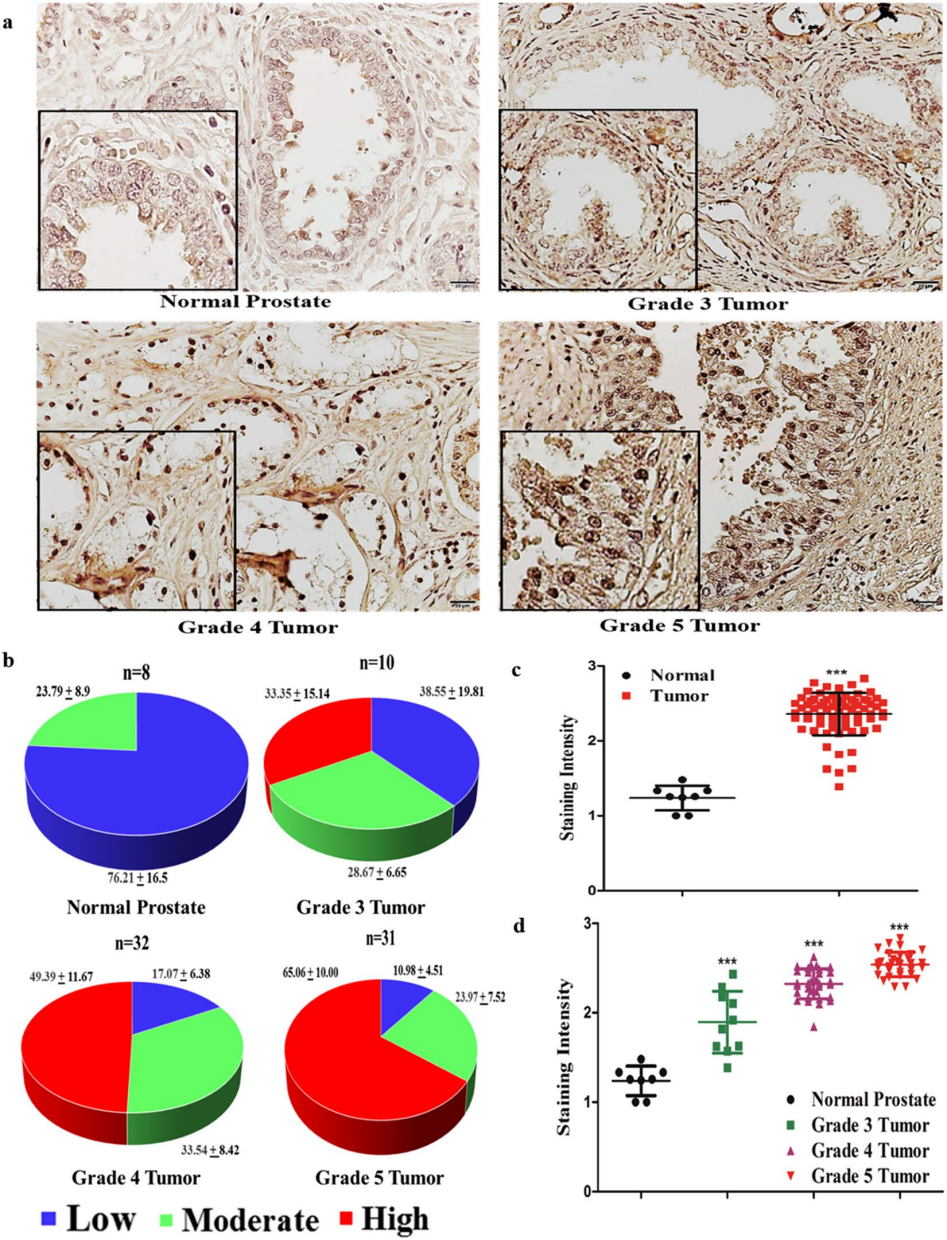
eIF4G1 protein expression is increased in prostate tissue sections of Prostate Cancer patients: Analysis of eIF4G1 expression in human prostate tissue microarray: (**a**) Representative photomicrograph image of eIF4G1 IHC staining on prostate tissue microarray with different grade tumors (40X). (**b**) Quantitation of eIF4G1 staining based on a high, moderate and low expression. (**c**) Staining intensity of eIF4G1 with normal prostate and tumor. (**d**) Staining intensity of eIF4G1 in the sample with normal prostate and different tumor grades. p-values are indicated as *<0.05, **<0.01, ***<0.001.

We further analyzed the eIF4G1 protein levels in a human prostate tissue microarray slide. Tissue sections from normal prostate expressed a lower level of eIF4G1 as compared to tissue sections from PCa. Moreover, we observed a graded increase in eIF4G1 protein as the disease progresses to an advanced stage. Analysis of TMA showed that 65% tissues showed high eIF4G1 expression in grade 5 prostate tumor samples and 49% of the tissues have high eIF4G1 staining in grade 4 tumor samples while with grade 3 tumor sample eIF4G1 expression was more or less evenly distributed for low, moderate and high (Fig. 2a,b). Quantitation data of staining intensity revealed that normal prostate tissues showed lower staining intensity compared to tumor samples (p < 0.0001) (Fig. 2c). Further our analysis of the eIF4G1 staining intensity based on tumor grade showed that normal prostate tissue has lowest staining intensity and with a graded increase in the tumor; staining intensity is increased statistically significant (normal prostate vs grade 3, 4, 5 prostate tumor p < 0.0001) (Fig. 2d). Taken together these data from TMA indicate that there is a significant positive correlation between expression of eIF4G1 and PCa aggressiveness.

### eIF4G1 is over-expressed in PCa cell lines and in tissue sections from TRAMP mice

**Figure 3:**
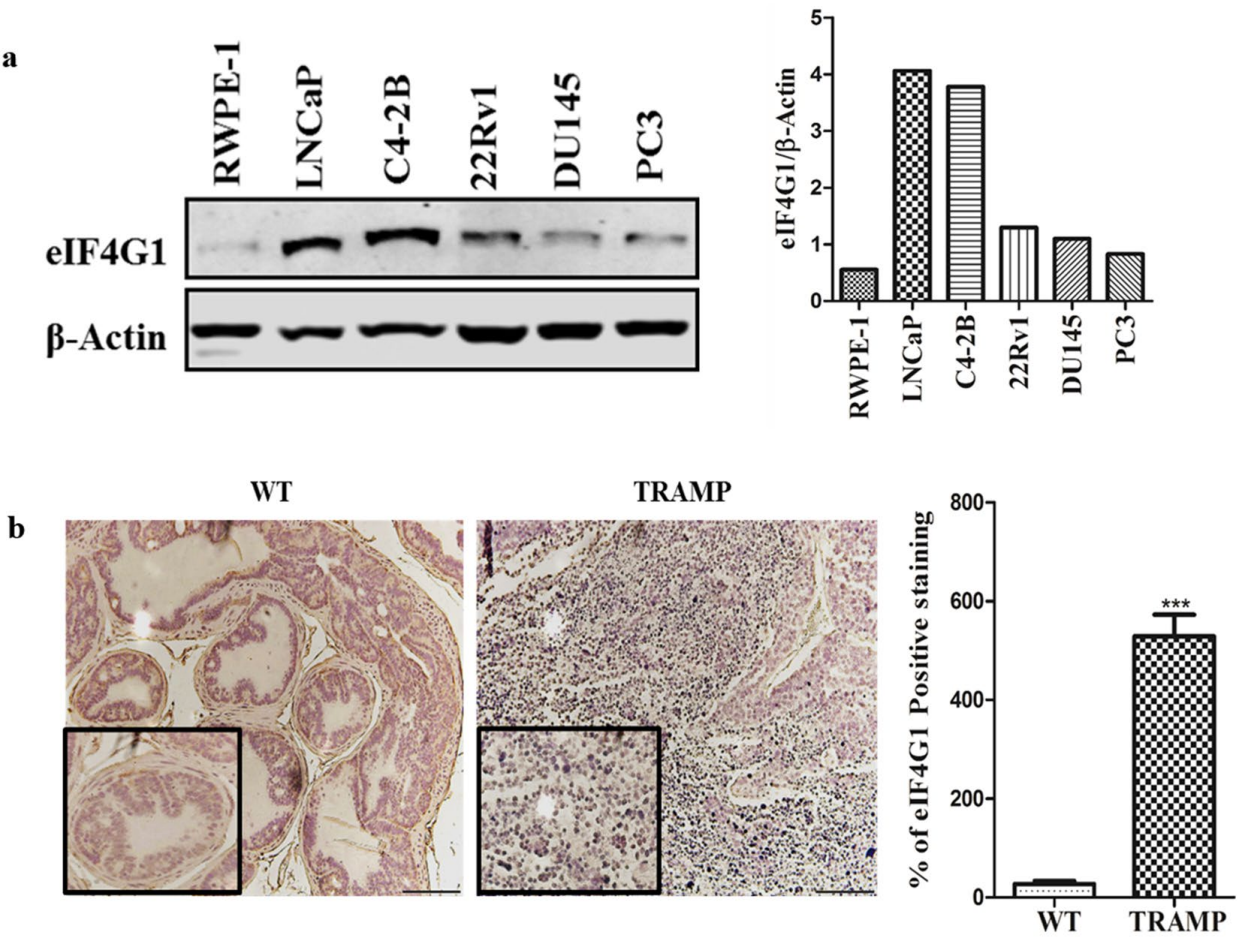
eIF4G1 expression is increased in PCa cell lines in comparison to normal cell line and during tumor progression in tissue sections from TRAMP mice: eIF4G1 expression in cell lines and tumor tissue: (**a**) eIF4G1 expression in PCa cell lines by Western Blot; (The adjacent graph is quantitation of western blot). (**b**) Photomicrograph of IHC for eIF4G1 expression on prostate tissue section from wild-type (WT) & 30 Week Old TRAMP mice (Images are X20, inset X40); (The Adjacent graph is quantitation). Error bars represents ± SD. p-values are indicated as *<0.05, **<0.01, ***<0.001.

To test the function of eIF4G1, we first analyzed the eIF4G1 protein levels in different PCa cell lines (LNCaP, C4-2B, 22Rv1, DU145, PC3) and in TRAMP prostate tumor tissues. We found that eIF4G1 protein expression was high in all PCa cell lines used in the present study when compared to normal human prostate cell line, RWPE-1 (Fig. 3a). Further, our immunohistochemistry analysis of eIF4G1 protein in 30-week old TRAMP prostate tumor tissues and wild-type (WT) prostate tissue revealed that eIF4G1 protein level was significantly (p < 0.0001) elevated in TRAMP prostate tissue as compared to WT prostate tissue (Fig. 3b). Taken collectively these data show that eIF4G1 expression was increased in cell culture models as well as the *in vivo* model system of PCa.

### Knockdown of eIF4G1 affects cell cycle-related genes and cell cycle progression

**Figure 4:**
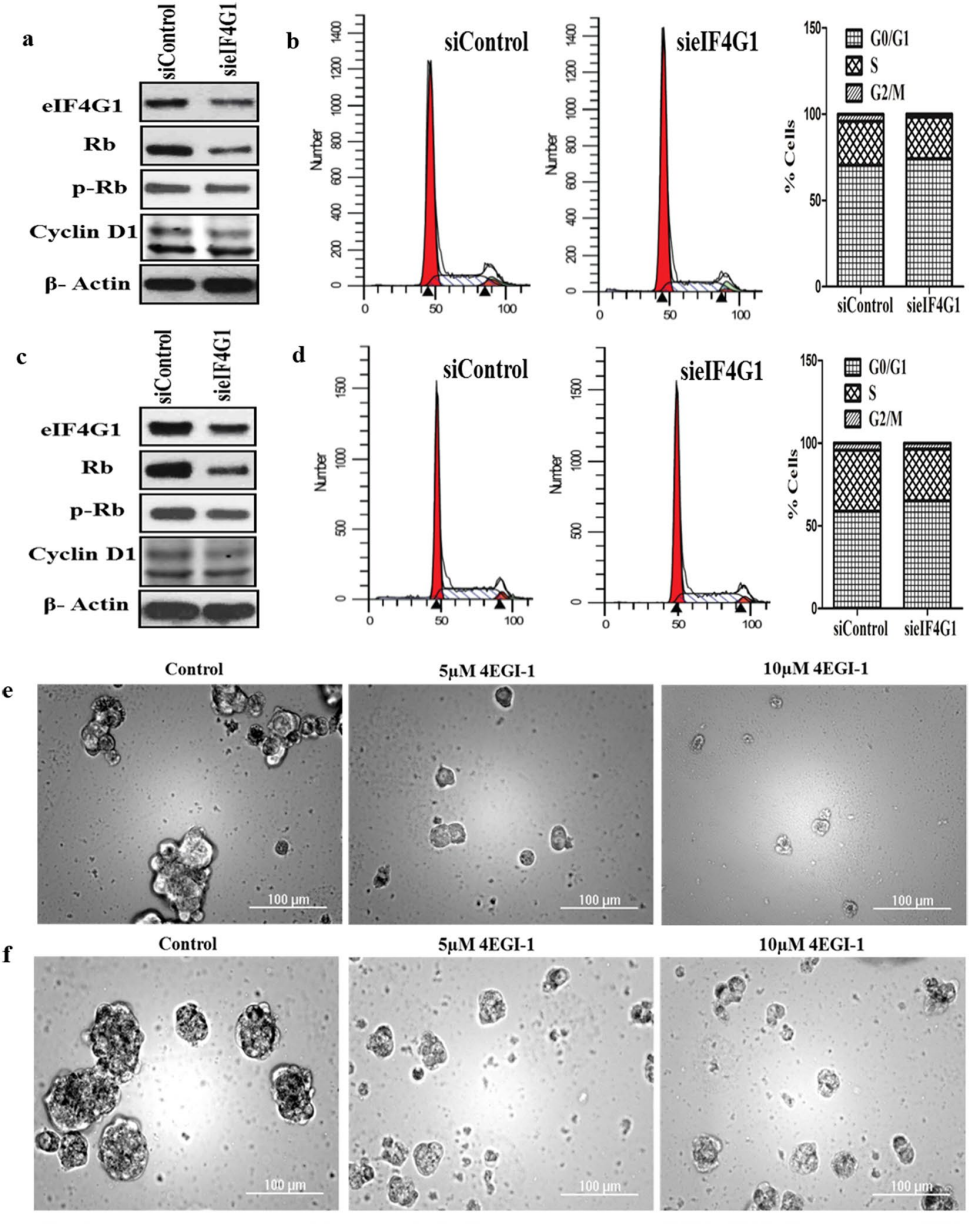
Knockdown of eIF4G1 reduces the levels of Cyclin D1, p-Rb, delays cell cycle and inhibitor of eIF4G complex impairs prostasphere formation: (**a**) Knockdown of eIF4G1 decreases Rb/p-Rb, Cyclin D1 protein levels and delays cells at the G0/G1 phase of cell cycle in LNCaP (**a**,**b**) and C4-2B (**c**,**d**). Adjacent is quantitation of cell cycle distribution in the different phase. (**e**). Representative images for prostasphere formation with control and treated with 5–10 µM of 4EGI-1 (eIF4G-eIF4E complex inhibitor) for LNCaP (**e**) and CRPC cells (C4-2B cells) (**f**).

Silencing of eIF4G1 affected several cell cycle/Checkpoint regulatory proteins, including Cyclin D1& p-Rb/Rb protein levels in LNCaP (Fig. 4a) as well as in C4-2B cells (Fig. 4c). We also observed the eIF4G1 knockdown resulted in a decrease in Cyclin D1 mRNA levels (Fig. S2). To further investigate the effects of eIF4G1 in PCa cell growth, we assessed the effect of the eIF4G1 knockdown on PCa cells, by cell cycle distribution. We found that silencing of eIF4G1 causes G0/G1 cell cycle delay in LNCaP (Fig. 4b) and C4-2B (Fig. 4d) cell lines. Taken jointly, our data suggested eIF4G1 played a role in the expression of the cell cycle-related proteins in PCa cells.

### eIF4G complex inhibitor impairs prostasphere formation

Further, we evaluated the functional role of eIF4G1 in LNCaP and C4-2B cells by using a known inhibitor of the eIF4G-eIF4E complex (4EGI-1) on LNCaP and CRPC cells (C4-2B cells) for prostasphere formation with control and treated with 05 or 10 µM of 4EGI-1 inhibitor. Treatment with inhibitor 4EGI-1 significantly impaired prostasphere formation in LNCaP (Fig. 4e) and in CRPC cells (C4-2B cells) (Fig. 4f), suggesting its functional role in prostasphere formation.

### eIF4G1 is required for PCa cell growth and colony formation

**Figure 5:**
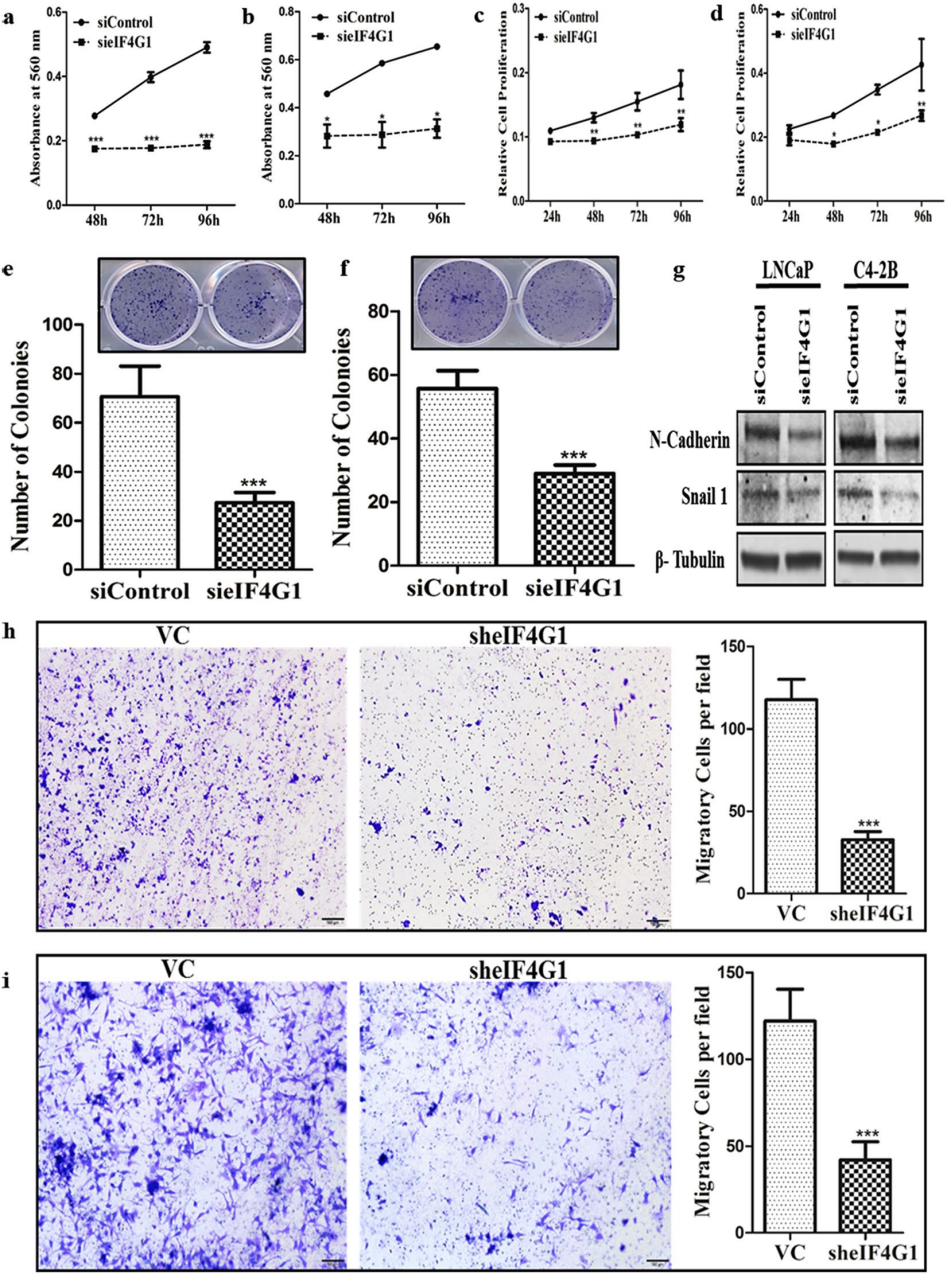
eIF4G1 is required for cell growth, colony formation, expression of EMT genes and cell migration in PCa Cells: Loss of Function studies following knockdown of eIF4G1 using siRNA (**a**,**b**). Cell viability was measured by crystal violet assay at 48–96 h with siControl and sieIF4G1, the graph showed absorbance at 560 nm for LNCaP (**a**) and C4-2B (**b**). (**c**) Cell proliferation assay for LNCaP and C4-2B (**d**) done by MTT assay at 24–96 h with siControl and sieIF4G1. (**e**) Colony formation for LNCaP and C4-2B (**f**) siControl and sieIF4G1, Lower panels: Colonies were counted using an ImageJ software. Error bar represents ± SD. (**g**) Representative image of immunoblotting for LNCaP and C4-2B for N-Cadherin & Snail 1 with siControl and sieIF4G1. (**h**) Representative image and graph of Transwell cell migration assays on LNCaP (**h**) & C4-2B (**i**) with Vector Control (VC) & sheIF4G1. p-values are indicated as *<0.05, **<0.01, ***<0.001.

Silencing of eIF4G1 (Fig. S4a) significantly impaired cell viability of LNCaP (Figs 5a, S3) and C4-2B (Figs 5b, S3) and proliferation of LNCaP (Fig. 5c) and C4-2B (Fig. 5d) cells measured at the various time point (24–96 h). We also observed that silencing of eIF4G1 effectively impaired the efficiency of colony formation of LNCaP (Fig. 5e) & C4-2B (Fig. 5f) cells. Taken together, these results showed that eIF4G1 is required for PCa cell growth and colony formation.

### Knockdown of eIF4G1 down-regulates EMT genes and limits PCa cell migration

Silencing of eIF4G1 (Fig. S4b) significantly decreased protein levels of EMT genes such as N-Cadherin and Snail 1 (Fig. 5g) and a significant decrease in mRNA levels of N-Cadherin, Snail 1 and Zeb 1 (Fig. S5). We also showed that silencing eIF4G1 in LNCaP (Fig. 5h) as well as C4-2B (Fig. 5i) cells resulted in a significant (p < 0.001) inhibition in cell migration. Taken together these results showed the important role of eIF4G1 in the expression of proteins required for EMT and the potential role of eIF4G1 in PCa cell migration.

### Knockdown of eIF4G1 impairs mRNA translation

**Figure 6:**
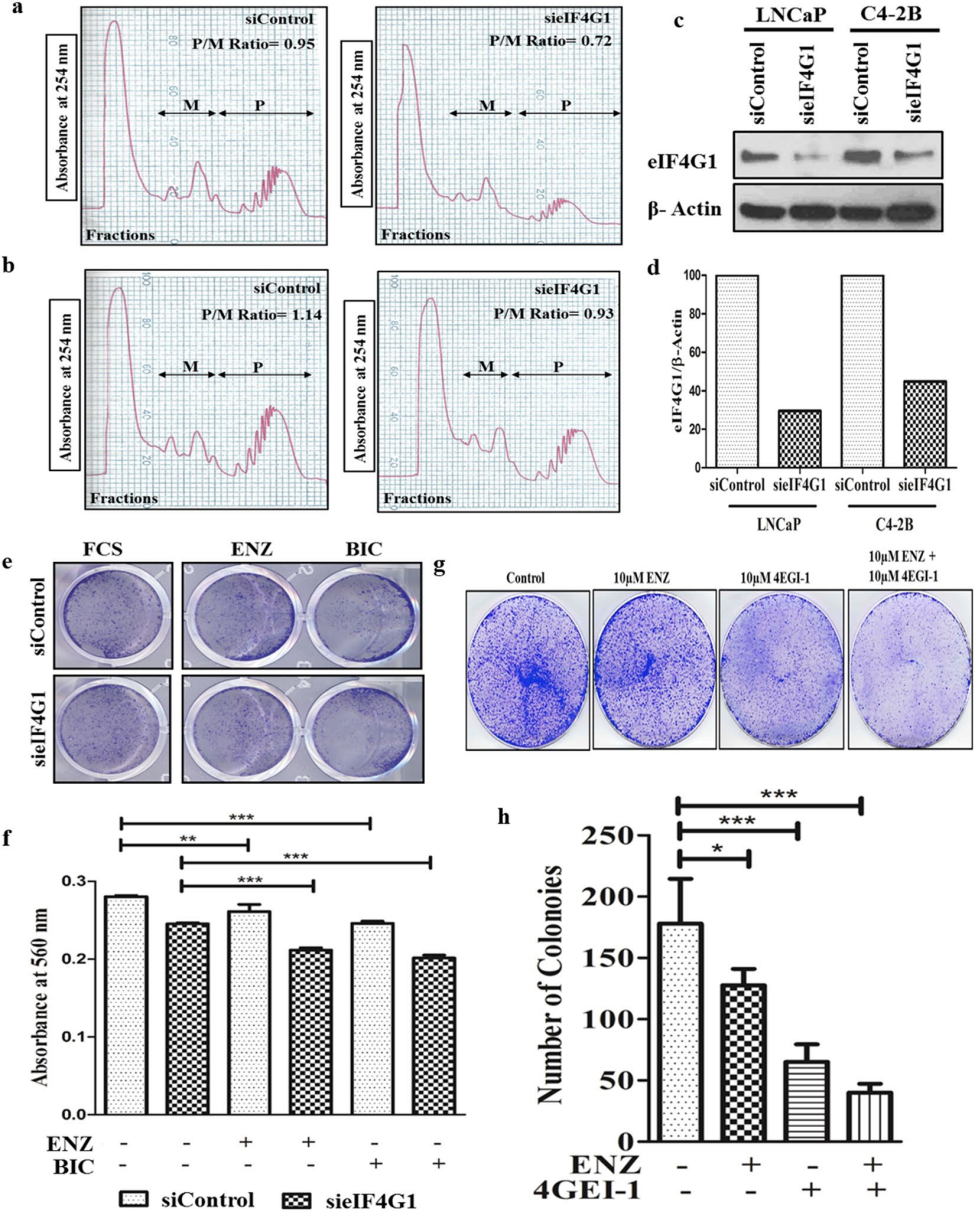
Knockdown of eIF4G1 impairs mRNA translation and sensitizes CRPC cells to current therapy and eIF4G complex inhibitor impairs colony formation in CRPC cells: Cytoplasmic extracts from siControl and sieIF4G1 for LNCaP (**a**) & C4-2B (**b**) cells were centrifuged respectively through continuous sucrose gradients to obtain fractions of increasing density. Absorbance (**A**_**254**_) (y-axis) versus increasing density (x-axis) of fractions was monitored for the presence of ribosomal subunits, ribosomes, and polysome. Polysome (P)/Monosome (M) Ratio was calculated further. (**c**) Western blotting showing knockdown of eIF4G1 in LNCaP and C4-2B. (**d**) The graph is quantitation of western blot for eIF4G1. (**e**) Representative image of cell viability assay done by crystal violet with CRPC (C4-2B cell line) cells with siControl and sieIF4G1 treated with 10 µM Enzalutamide (ENZ) and 10 µM Bicalutamide (BIC) for 24 h. (**f**) The Graph showed quantitation for above plate absorbance at 560 nm. (**g**) Colony formation assay for CRPC cells (C4-2B) with 10 µM ENZ, 10 µM 4EGI-1 (eIF4G-eIF4E complex inhibitor) and combination of 10 µM 4EGI-1 and 10 µM ENZ treatment. (**h**) The graph is the number of colonies counted. Colonies were counted using an ImageJ software. Error bars represent ± SD. p-values are indicated as *<0.05, **<0.01, ***<0.001.

We evaluated mRNA loading to polysomes using Polysome profiles generated from LNCaP & C4-2B cells with the following siRNA mediated knockdown of eIF4G1. Results (Fig. 6c) showed western blot for eIF4G1 in siControl and sieIF4G1 knockdown cells that were used for generating polysome distribution profile. We observed a decrease in mRNA (based on absorbance at 254 nm) associated with polysome peaks and a concomitant increase in mRNA association with monosome fractions in eIF4G1 cells compared to respective controls. For the global translation activity, estimation of Polysome-to-Monosome (P/M) ratio was determined. There was a change in P/M ratio (siControl 0.95 vs sieIF4G1 0.72) in LNCaP cells (Fig. 6a) as well as (siControl 1.14 vs sieIF4G1 0.93) in C4-2B cells (Fig. 6b). These results suggest that eIF4G1 plays an important role in translation initiation step in PCa cells.

### Knockdown of eIF4G1 sensitize CRPC cells to Enzalutamide and Bicalutamide

We further also evaluated the effects of eIF4G1 knockdown on the sensitivity of CRPC (C4-2B) cells to Enzalutamide (ENZ) and Bicalutamide (BIC). For these studies, Control and eIF4G1 knockdown cells were grown in the presence or absence of 10 µM ENZ or 10 µM BIC. Results (Fig. 6e,f) showed that ENZ and BIC treatment resulted in significantly decreased cell numbers in eIF4G1 knockdown cells as compared to their respective controls. These results suggested that eIF4G1 knockdown sensitized CRPC cells (C4-2B cells) to ENZ as well as BIC, suggesting the eIF4G1 could serve as a potential therapeutic target for sensitizing CRPC cells to the current treatment regimen.

### eIF4G complex inhibitor impairs colony formation in C4-2B cells

Further, we checked the effects of the known inhibitor of the eIF4G-eIF4E complex (4EGI-1) on CRPC cells (C4-2B cells) in combination with present treatment available on colony formation. We treated CRPC cells with 10 µM ENZ, 10 µM 4EGI-1 and combination of 10 µM 4EGI-1 and 10 µM ENZ. Colony formation in C4-2B cells (Fig. 6g,h) was impaired with treatment with 4EGI-1 as well as in combination with Enzalutamide (ENZ).

## Discussion

Current therapies for metastatic CRPC are ineffective as such CRPC results in over 25,000 deaths in the USA each year. eIF4G1 has been shown to be over-expressed in several malignancies, where it has been shown to play an important role in oncogenic properties^12–19^. In a very recent study in 2017, on 398 cancer cells revealed that eIF4G1 is essential to cancer cell survival^21^. In another study, showed that phage clones representing known protein for eIF4G1 were substantially more reactive with serum from patients with PCa than with that from controls^22^. To the best of our knowledge, this is the first study to investigate the role of eIF4G1 in PCa. Here we report for the first time, the expression of eIF4G1 is significantly increased in PCa cell lines and tumor tissues and in multiple clinical cohorts as compared to respective controls. We also show for the first time a positive correlation of eIF4G1 expression with the PCa progression.

Prostate cancer is a heterogeneous disease, with disease manifestations ranging from in a majority of patients as an indolent disease of no significant consequence, to patients health, to a rapid and many times fatal progression. Elevated levels of serum PSA (Prostate Specific Antigen) remain the gold standard, yet a controversial method for early detection of prostate cancer. Current diagnostic methods do not differentiate men whose tumors require immediate and aggressive therapy from those that might require just clinical observation. As a result, many patients, with otherwise indolent prostate cancer have to suffer unnecessary treatments, while others die from aggressive disease diagnosed too late^23^. Our results suggest that eIF4G1 expression may help stratify PCa patients and may serve as a tumor marker for distinguishing indolent disease from lethal PCa.

Therapeutic utility of targeting eIF4G1 and disrupt select pathways in mRNA translation lies within its strength to affect the expression of multiple oncogenic pathways that are associated with disease and progression and rely on cap-dependent translation. In the present study, we observed that knocking down of eIF4G1 results in cell cycle delay in G0/G1 phase. Deregulated cell growth and proliferation is the hallmark of cancer cells including in PCa^24,25^. Multiple oncogenic drivers target cell cycle regulatory machinery resulting in uncontrolled cell proliferation as such targeted agents have had a limited success in halting cell cycle and limiting tumor growth. Our results suggest that eIF4G1 could serve as a novel therapeutic target for disrupting uncontrolled growth and proliferation of PCa and perhaps other malignancies.

Almost all PCa deaths result from tumor metastasis to the distant organs and emergence of CRPC phenotype and treatment resistance. Epithelial-to-mesenchymal transition (EMT) is required for cancer cells to metastasize to the distant organs^26^. We observed that silencing of eIF4G1 resulted in inhibition of genes associated with EMT, limited cell migration and clonogenic activity of PCa cells. We also observed that silencing eIF4G1 increased sensitivity of CRPC cells to Bicalutamide and Enzalutamide. EMT is also known to associate with treatment resistance^27^. As such identifying new therapeutic targets to overcome EMT associated resistance is a major therapeutic goal.

For advanced PCa first line of treatment is Androgen depletion therapy (ADT). When ADT fails, it leads to castration-resistant or recurrent prostate cancer (CRPC)^2^. Next generation AR inhibitors temporary halt PCa progression and in due course resistance develops and the disease progresses^28,29^. In light of these observations, our results suggest that eIF4G1 may serve as a novel target for intervention in CRPC. Considering our findings for eIF4G1 in PCa, the available inhibitor of eIF4E-eIF4G complex i.e. 4EGI-1^30^ can be used in combination with ENZ that may sensitize the CRPC cells to current therapy and improve overall survival and disease outcome.

**Figure 7:**
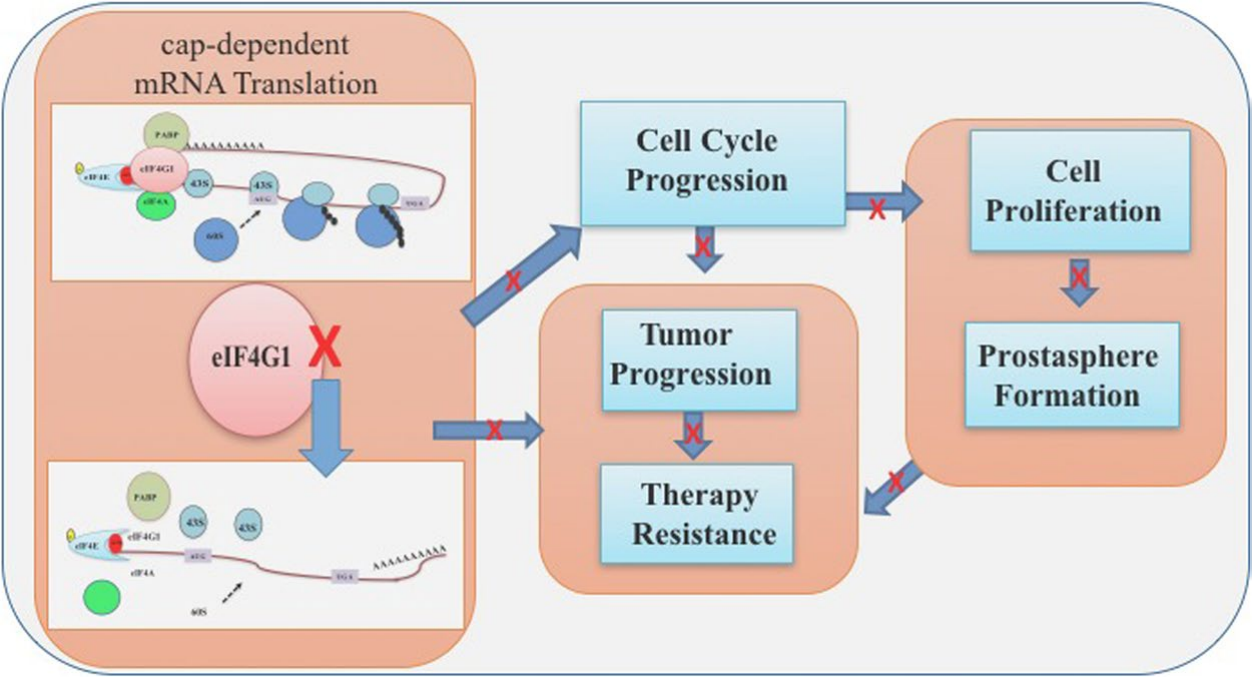
Model for eIF4G1 regulated network in PCa.

Our findings support the hypothesis of combinatorial therapy concept by using eIF4G1 as a novel target in PCa patients with therapy resistance. Overall, our data indicate that eIF4G1 may function as an oncoprotein in PCa and serve as a new diagnostic and/or prognostic marker in PCa, and a potential therapeutic target for intervention in CRPC (Fig. 7).

## Materials and Methods

### Materials

Antibodies for eIF4G1 (Cell Signaling #2858); Cyclin D1 (Santa Cruz sc-20044); Rb (Cell Signaling #9309); p-Rb (Cell Signaling #9308); β-Tubulin (Developmental Studies Hybridoma Bank E7); β-Actin (Sigma A2228) were used to probe respective proteins of interest. Inhibitor for eIF4G-eIF4E complex (4EGI-1), (Catalog No. S7369) were purchased from Selleck Chemicals.

### Cell Line/Culture

Cell lines LNCaP (CRL-1740), 22Rv1 (CRL-2505), DU145 (HTB-81), PC3 (CRL-1435) and RWPE-1 (CRL-11609) were purchased from ATCC and C4-2B cell line was gift from Dr. Leland Chung’s lab. LNCaP and 22Rv1 cells were cultured in RPMI 1640 complete medium (Hyclone: Cat No.: 30255.01) and PC3 and DU145 cells were cultured in DMEM/F12 and DMEM with 10% v/v Fetal Bovine Serum, 1% v/v Antibiotics (Penicillin and Streptomycin) respectively. The normal Prostate cell line RWPE-1 was cultured in Keratinocyte-SFM (Thermo Fisher Cat No.: 17005042) with EGF 1–53 (Epidermal Growth Factor 1–53) and BPE (Bovine Pituitary Extract). The cells were incubated at 37 °C in a 5% CO_2_ humidified atmosphere. All cell lines were authenticated by using Short Tandem Repeat (STR) analysis service by ATCC (135XV3). We also tested the cell lines for the presence of Mycoplasma by Mycoplasma PCR detection kit (abm cat. No. G238).

### Animal Studies

We used the Prostate tumor tissue sections from the 30 Week old Transgenic Adenocarcinoma of the Mouse Prostate (TRAMP) model, which mimics the disease phenotype of human prostate cancer. Male TRAMP mice spontaneously develop prostate tumors following the onset of puberty. The animal studies were approved by LSU Health Sciences Center at Shreveport Animal Care and Use Committee (LSUHSC-S IACUC) and all the methods were performed in accordance with the relevant guidelines and regulations.

### TCGA data mining and retrieval of data from the clinical data set

mRNA expression and clinical data for eIF4G1 and eIF4E from TCGA (The Cancer Genome Atlas)^31^ data set for the prostate cancer and normal samples were analyzed by UALCAN (http://ualcan.path.uab.edu/)^32^ web server and TCGA database. The analysis was done on 497 primary tumors of prostate cancer and 52 normal samples from TCGA. Using a gene name (or more), the web server will mine the data available for the gene expression with cancer stage, Gleason score and survival analysis and statistical significant p-value for each group/subgroup analysis. To study gene expression changes from the different clinical dataset, we used freely accessible cBioPortal (http://www.cbioportal.org) tool^33,34^. All prostate tumors with mRNA expression data from the Metastatic Prostate Cancer dataset^35^ and Neuroendocrine Prostate Cancer dataset (n = 114)^36^ with a mRNA Z-score threshold of ±2 as compared with normal prostate samples were used for eIF4G1. Genetic alterations in percent mRNA up-regulation and amplification were taken into consideration for the present study.

### Human Prostate tissue Microarray and Immunohistochemistry (IHC)

Tissue Microarray (TMA) for prostate (US Biomax, Inc. #PR1921a) was used to evaluate the eIF4G1 protein levels in the patient samples. This TMA contains 80 cases of adenocarcinoma, 8 adjacent normal prostate tissues and 8 normal prostate tissues. Immunohistochemistry for eIF4G1 on TMA and prostate tissue sections from wild-type (WT) mice, as well as the 30-week old TRAMP prostate tumor tissue was performed as described^37^. Photomicrographs for representative tissue sections were taken by Olympus BX51 microscope at X20 and X40 magnification.

Expression of eIF4G1 was considered high if over 60% fields showed positive staining, expression was moderate if expression ranged from 40 to 60% positive eIF4G1 staining and expression were counted as low if less than 40% area showed positive eIF4G1 staining as described^37^.

### Western blot analysis

Western blot analysis was performed as described^38^ for the respective protein. Blots were scanned for the respective protein of interest and loading control either by LI-COR Odyssey CLx (LI-COR, Lincoln, USA) system by using IRDye 680 goat anti-mouse/IRDye 800 goat anti-rabbit secondary antibodies or HRP conjugated secondary mouse/rabbit and developed by the film.

### siRNA/shRNA Mediated eIF4G1 knockdown

For the loss of function studies, we knockdown eIF4G1 in cells, we used siRNA for eIF4G1 (human) from Santa Cruz (sc-35286) and non-targeting siRNA-A as a negative control from Santa Cruz (sc-37007). Where indicated we used shRNA vector control (SHC001) (VC) and shRNA for eIF4G1 (SHCLNG-NM_182917, TRCN0000061770) from Sigma-Aldrich. Transfection of this siRNA/shRNA was performed in six-well plates using the HiPerFect transfection reagent (Qiagen, CA)/Lipofectamine 2000 reagents (Life Technologies, Invitrogen) respectively as per manufacturer’s protocol. Effect of knockdown was checked by measuring protein levels for eIF4G1 by western blot assay.

### Flow cytometry

Cell Cycle Analysis was performed as described previously^39^. Cell cycle distributions were analyzed by using BD LSR II Flow Cytometer at Core Facility of the LSU Health Sciences Center, Shreveport and ModFit LT Software was used for analysis.

### Real-Time (RT)-PCR

Total RNA was extracted using a commercially available RNA isolation kit (OMEGA), followed by cDNA synthesis using iScript cDNA synthesis kit (Bio-Rad). Further RT-PCR was done as described previously^40^ for eIF4G1, Cyclin D1, N-Cadherin, Vimentin, Snail 1 and Zeb 1 by using respective RT-PCR primer sequence (Supplementary Table 1).

### Cell proliferation and viability

Cell proliferation and viability assays were performed by MTT assay and Crystal Violet staining respectively as described previously^39^.

### Clonogenic Assay

Clonogenic activity was measured as described previously^39^ for LNCaP and C4-2B and visible colonies were counted by NIH ImageJ software.

### Cell Migration Assay

Trans-well cell migration assay was done for vector control and sheIF4G1 in LNCaP and C4-2B cells as mentioned^41^. Images of migratory cells on the underside of the trans-well were captured using Olympus BX51 microscope at 10X magnification. The migratory cells were counted by counting four fields per stained membrane.

### Prostate tumor sphere (Prostasphere) formation

Prostasphere formation was done for LNCaP and C4-2B cells as described^42^ with some modifications. Briefly, LNCaP and C4-2B cells were trypsinized, counted and seeded at 2000 cells per 35 mm ultra-low attachment dish in spheroid media from PromoCell (Cat. No. C-28070). Minimum 5 adhered cells were considered as spheroid growth. Images were captured with Cytation5 image reader (BioTeck) at 10X magnification.

### Polysome profiling

Polysome-bound RNA fractionation was performed as described^43^ with modifications. 10 × 10^6^ cells for LNCaP and C4-2B were used in each assay. Briefly, before harvesting, cells were pulsed with 100 µg/ml of Cycloheximide for 10 minutes. And lysed in PL Buffer (Polysome lysis) containing 20 mmol/L Tris-HCl (pH 7.5), 250 mmol/L NaCl, 15 mmol/L MgCl2, 0.5% NP-40, 100 µg/mL Cycloheximide, 2 mmol/L DTT, 50 µg/mL heparin, and 200 U/mL RNasin (Promega) and homogenized. Cell lysates were centrifuged and the supernatant was loaded onto a 10% to 60% sucrose gradient tube. Tubes were centrifuged at 35,000 g for 3 hours at 4 °C and fractions were collected using Density Gradient Fractionation System by ISCO with continuous monitoring based on an absorbance at 254 nm. To calculate polysome to monosome ratio graph were scanned and pixels of polysome and monosome were measured with tpsUtil64 and tpsDIG2w64 software. (http://life.bio.sunysb.edu/morph/).

### Statistical analysis

Data are expressed as means ± standard deviations (SD). The two-tailed Student t-test and ANOVA test were used for statistical analysis of experiments and GraphPad Prism5 was used for statistical analysis. Significant differences in p-values are showed as *<0.05, **<0.01, ***<0.001.

### Availability of data and material

All data generated or analyzed during this study are included either in this article or in the supplementary files.

## Electronic supplementary material

Supplementary Data
