# Supplementary material for: Eukaryotic Translation Initiation Factor 4 Gamma 1 (eIF4G1) is upregulated during Prostate cancer progression and modulates cell growth and metastasis
Source: Sci Rep. 2018 May 10;8:7459. doi: 10.1038/s41598-018-25798-7 (PMC5945649; doi:10.1038/s41598-018-25798-7)
Supplement: Supplementary file 1 — Supplementary Data [file 41598_2018_25798_MOESM1_ESM.pdf]

Supplementary data:

Eukaryotic Translation Initiation Factor 4 Gamma 1 (eIF4G1) is upregulated during Prostate cancer progression and modulates cell growth and metastasis

Praveen Kumar Jaiswal[1], Sweaty Koul[2,4], Prakash Srinivasan Timiri Shanmugam[1] and Hari K. Koul[1, 3, 4¶]

[1]Department of Biochemistry and Molecular Biology; [2]Department of Urology; LSU Health Sciences-Center-Shreveport, LA; [3]Overton Brooks Veterans Administration Medical Center, Shreveport, LA, and [4]Feist Weiller Cancer Center, Shreveport, 1501 Kings Highway; LA-71130.

[¶]**Corresponding Author:** Dr. Hari K. Koul, Professor, Carrol W. Feist Chair, Dept. of Biochemistry and Molecular Biology, LSU Health Sciences Center, 1501 Kings Highway, PO Box 33932, Shreveport, LA 71130-3932. hkoul@lsuhsc.edu

Running Title: Functional Role of eIF4G1 in Prostate Cancer

S1a

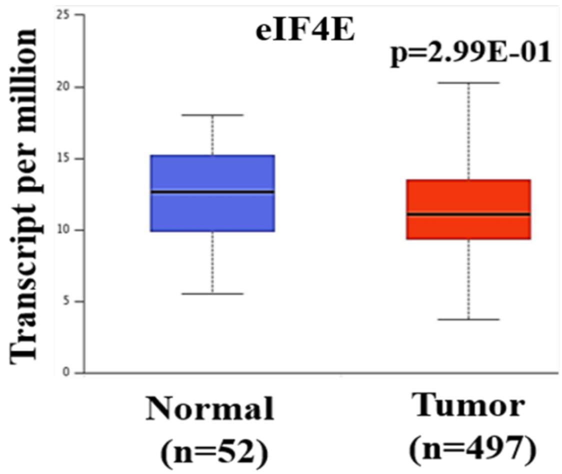

S1b

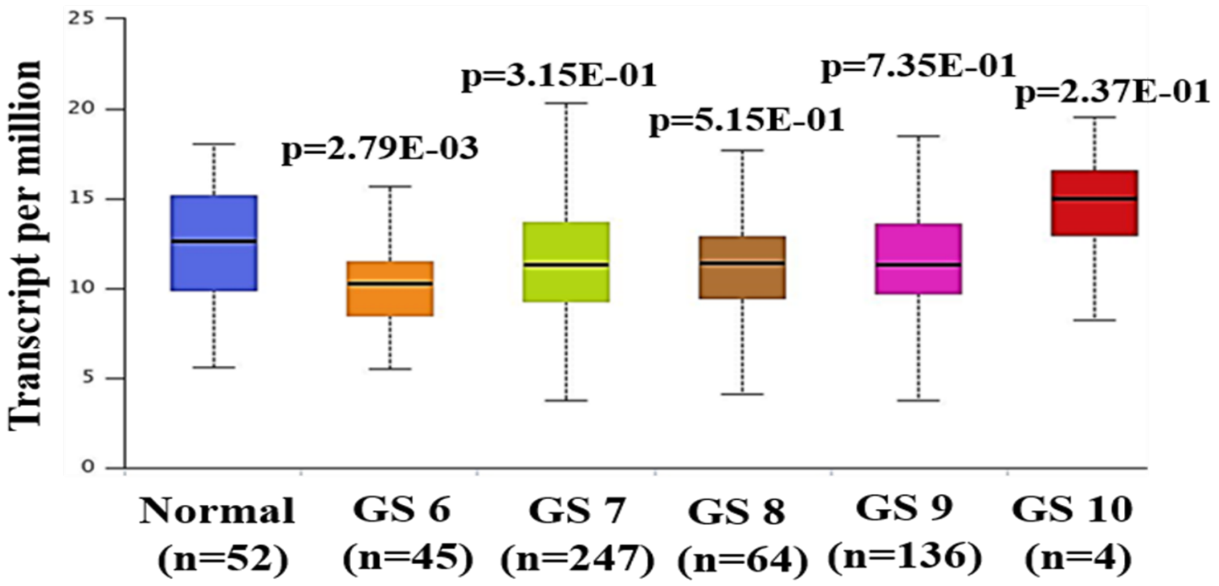

Figure S1: Analysis of eIF4E mRNA expression in PCa samples from TCGA database:
a. mRNA levels of eIF4E in Normal vs prostate tumor samples. **b.** eIF4E with increasing Gleason Score (GS) with statistically insignificant p-value except with GS 6.

S2

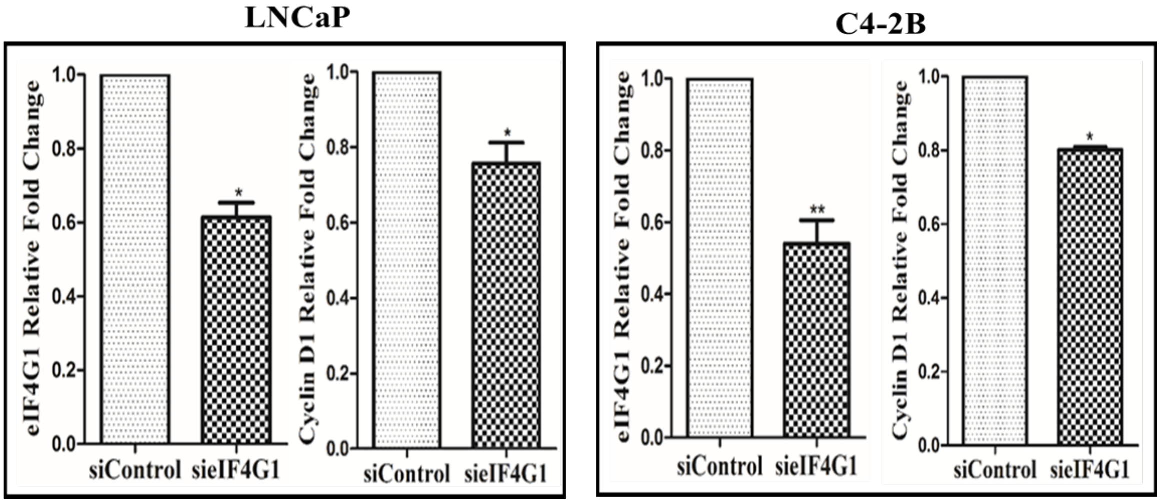

Figure S2: Knockdown of eIF4G1 decreases mRNA level of eIF4G1 & Cyclin D1: Knockdown of eIF4G1 decreases mRNA level of eIF4G1 & Cyclin D1 in LNCaP and C4-2B with si Control/si eIF4G1. p-values are indicated as *<0.05, **<0.01, ***<0.001.

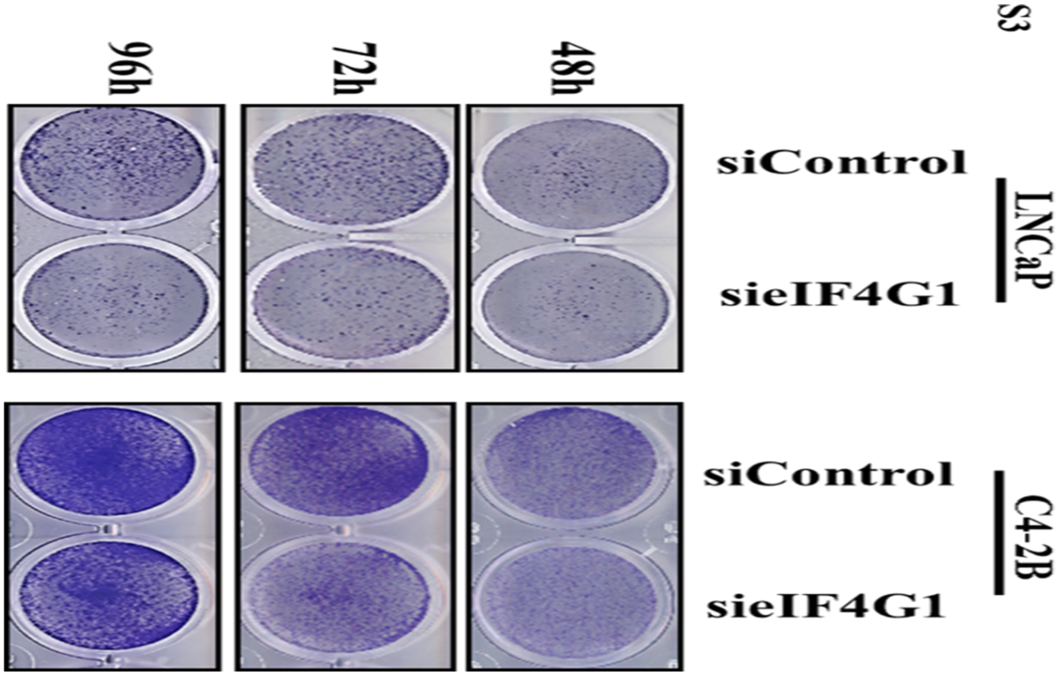

Figure S3: eIF4G1 promotes cell viability: Representative image of cell viability assay done by crystal violet in LNCaP and C4-2B with si Control and si eIF4G1 at 48/72/96h.

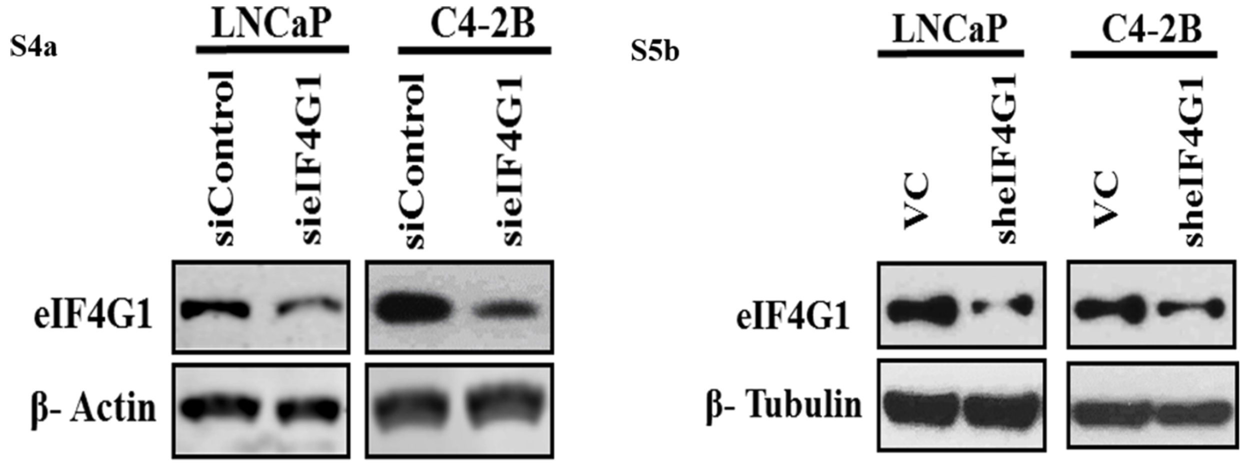

Figure S4: Western blot showing knockdown of eIF4G1: Knockdown of eIF4G1 decreases eIF4G1 protein in LNCaP and C4-2B with si Control/si eIF4G1 (S4a) and with VC/sheIF4G1 (S4b).

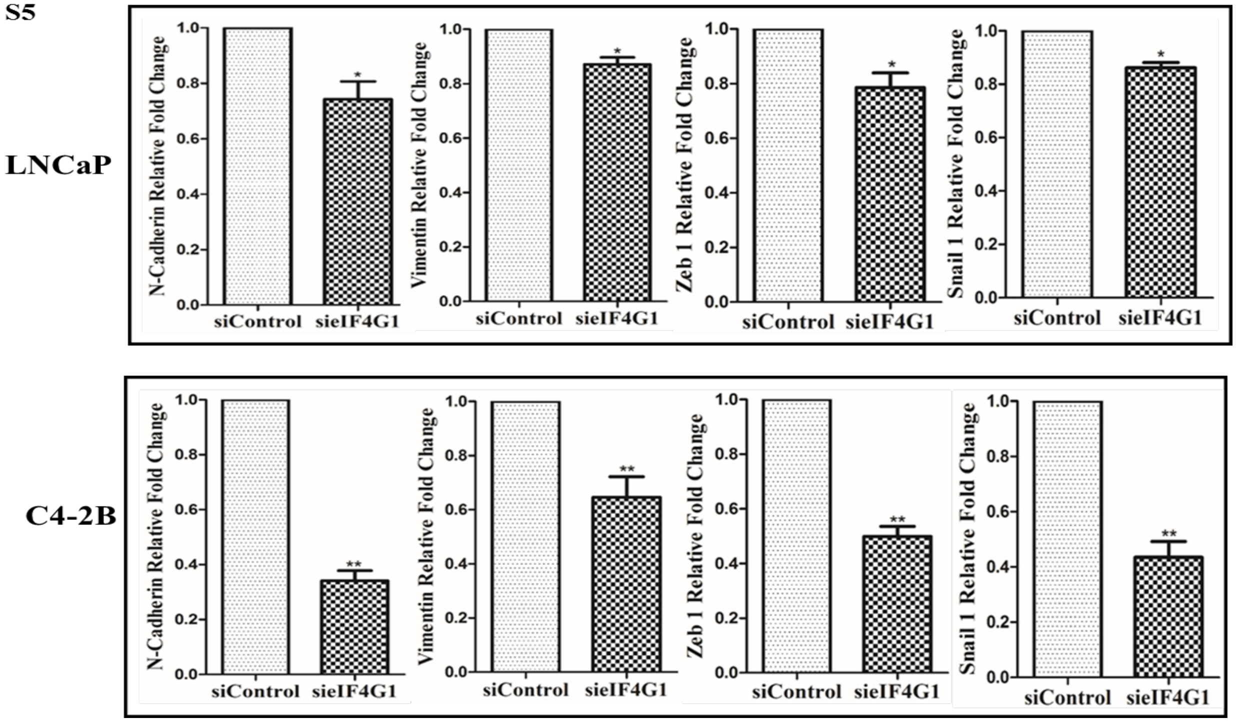

Figure S5: RT-PCR data for EMT genes on si Control and si eIF4G1 in LNCaP & C4-2B cells: Real-time PCR data showing Knockdown of eIF4G1 decreases EMT genes i.e. N-Cadherin, Vimentin, Zeb 1 & Snail 1 in LNCaP and C4-2B with si Control and sieIF4G1. p-values are indicated as *<0.05, **<0.01, ***<0.001.

**Table S1: Primer sequence used for RT-PCR**	
**Gene**	**Primer Sequence**
**eIF4G1**	Forward 5’ TTGTGGATGATGGTGGCT 3’
	Reverse 5’ TTATCTGTGCTTTCTGTGGGT 3’
**Cyclin D1**	Forward 5’ GCGGAGGAGAACAAACAGAT 3’
	Reverse 5’ GAGGGCGGATTGGAAATGA 3’
**GAPDH**	Forward 5’ GAGTCAACGGATTTGGTCGT 3’
	Reverse 5’ TTGATTTTGGAGGGATCTCG 3’
**N-Cadherin**	Forward 5’ GCTCCCTTAATTCCTCAAGTAG 3’
	Reverse 5’ TCATCACCTCCACCATACA 3’
**Snail 1**	Forward 5’ GACCACTATGCCGCGCTCTT 3’
	Reverse 5’ TCGCTGTAGTTAGGCTTCCGATT 3’
**Vimentin**	Forward 5’ TGAGTACCGGAGACAGGTGCAG 3’
	Reverse 5’ TAGCAGCTTCAACGGCAAAGTTC 3’
**Zeb 1**	Forward 5’ GCACAACCAAGTGCAGAAGA 3’
	Reverse 5’ CATTTGCAGATTGAGGCTGA 3’
